# Epidemiology of Home-Related Injuries During a Six-Year Period in Kashan, Iran

**DOI:** 10.5812/atr.7709

**Published:** 2012-10-14

**Authors:** Mohammad Reza Fazel, Esmaeil Fakharian, Ebrahim Razi, Masoumeh Abedzadeh-Kalahroudi, Mehrdad Mahdian, Mahdi Mohammadzadeh, Mohaddeseh Pourpooneh

**Affiliations:** 1 Trauma Research Center, Kashan University of Medical Sciences, Kashan, IR Iran; 2 Student Research Committee, Kashan University of Medical Sciences, Kashan, IR Iran

**Keywords:** Epidemiology, Home, Injury

## Abstract

**Background:**

Injury is one of the leading causes of morbidity and mortality in the world, and the home is one of the most common places for these types of injuries.

**Objectives:**

This study is designed to investigate the epidemiology of home-related injuries in Kashan, Iran.

**Patients and Methods:**

This investigation is a retrospective cross-sectional study on existing data from the data bank of the Trauma Research Center at Kashan University of Medical Sciences during a six-year period. Demographic data such as; sex, age, place of residence, educational and occupational status, injury mechanism, injured organs and injury outcomes, were analyzed using a chi-squared test and P < 0.05 was considered significant.

**Results:**

The number of home injuries was 10146 in total, that included about 25.2% of all injuries in Kashan City. Most of the injured people were men (58.3%), 87.4% lived in the city and 18.6% were aged more than 64 years. The majority (42.7%) had a primary or secondary school education and 27.2% were housewives. Falling from a height was the most common cause of injury (55.3%). Limbs were the most common body region that was injured (73.7%). Young men (under 15 years) and older women (over 65 years) had more injuries, especially from falls. There was a statistically significant difference between the sex and age of the injured people (P < 0.001), sex and injury mechanism (P < 0.001), and also between the injury mechanism and sex in the age groups (P < 0.001).

**Conclusions:**

The most common injury mechanism in regard to home accidents was falls; therefore fall-related injury prevention programs should be designed to make homes safer and education should focus on changes in lifestyle to reduce fall susceptibility.

## 1. Background

Injury is one of the leading causes of morbidity and mortality in the world and all communities regardless of income or geographic region are involved with accidents (1). Approximately 5.7 million people died from injuries in 2004 and that included 9.8% of all causes of death (2). It has been estimated that every day 16 000 person die from injuries (1). In the world, about 90% of the burden of injuries are related to low and middle-income countries (3). In these countries, injuries are among the most common causes of death in the 15-59 age group and men have a higher death rate than women (2). A study in Iran showed that 28% of the total number of Disability Adjusted Life Years (DALYs) was due to injuries; including 1.071 million for men and 235000 for women. The highest rate of mortality in the 5–44 age group was related to injuries (4). In the USA, the four leading causes of medically-treated injury in 2010 were; falls, overexertion, being struck by a person or an object and traffic accidents (5, 6). One of the important places for injury is the home. Approximately a fifth of all unintentional injuries occurs in the home environment (6). In the USA, 34.9 million episodes of injury and poisoning occurred in 2010, and about 50% of these cases were in and around the home (5). In England and Wales, the number of deaths due to injuries inside and around the home was approximately in the age groups case (7). In some low and middle-income countries, most of the injury related studies have focused on traffic injuries (8, 9) and little attention has been given to home injuries. Several studies in Iran have shown that home accidents were the second highest cause of injuries after traffic accidents (10-12). Results of a study by Khosravi on home accidents showed that most of the injured people were men (53.2%) and the three leading causes of injury were; burns, injuries due to sharp objects and falls (13). Another survey in Shiraz City showed a mortality rate of 1.3% for home injuries. Also injuries were more common in women (52.1%) than in men (47.9%); urban (66.3%) rather than rural areas (33.7%) and in children aged less than 5 years. The leading causes of injuries were; burns (66.5%) and injuries due to sharp objects (11.3%) (14). A study on home-related injuries in Iran revealed that injury rates among children aged 0 – 4 years were the highest and among the elderly (≥ 60 years) were lowest. This prevalence varied between the sexes; most patients in age groups under 15 years were male, but in all age groups more than 15 years, most of the patients were female. Burns, falls and poisoning were the three most common causes of death (15). Home accidents are preventable, but based on population characteristics and the type of accident, their managements are different.

## 2. Objectives

This study was designed to investigate the epidemiology of home-related injuries in trauma patients treated at the Shahid Beheshti hospital in Kashan city during a six-year period. 

## 3. Patients and Methods 

This investigation is a cross-sectional, retrospective study on existing data from the data bank of the Trauma Research Center in Kashan University of Medical Sciences over a six-year period. Kashan is located in the central part of Iran, with a population of approximately 400 000. The Trauma Research Center has gathered information of all injuries that required medical attention since 2004. For the purpose of this study, the entire of home injury patients that were treated at the Shahid Beheshti hospital, from 2005 to 2010 was reviewed. Exclusion criteria included; patients with incomplete records, poisonings and burns, because patients with these types of injuries may be directly admitted to burn units or other inpatient units, therefore their data may not be entry on the data bank of the Trauma Research Center. Demographic data such as; sex, age, place of residence, educational and occupational status, and injury data including; mechanism of injury, injured organs and injury outcomes were analyzed using a chi-square test and P < 0.05 considered significant.

## 4. Results 

The total number of all injuries during this period was 40277 and the number of home injuries was 10146, and that included about 25.2% of all injuries in Kashan City. Table 1 presents the characteristics of the injured patients. The majority of the patients were men (58.3%). Male/Female ratio for home injuries was 1.39. The majority of the injured people (87.4%) lived in the city; 18.6% were aged more than 64, and 30.6% were aged under 15 years. The majority (42.7%) had primary or secondary school education and 27.2% were housewives. Overall, 58.3% and 41.7% of the home injuries occurred in men and women respectively, the incidence rate was different for the age groups of both sexes. Boys had more injuries than girls (33.3% versus 26.9%) in the age group under 15 years, but in adults at the age of 55 years or more, women had more injuries than men, 60.3% of the reported injuries were in women 55–64, and 62.3% in women older than 65 years compared with 39.7% in men 55-64, and 37.7% for the men over the age of 65. There was a statistically significant difference between the sex and age of the injured people (P = 0.000). Fall injuries were the most common injury mechanism (70.4%), followed by sharp-object injuries (15.1%), 38.9% of fall injuries related to falls from a height, 12.8% falls from stairs, 3.6% falls from a bed or tree and 15.1% for falls on the same level. Table 2 shows that fall injuries are higher in women than men (77% for women versus 65.5% for men), but for other mechanisms of injury (sharp objects and conflict) men experience higher rates than women.

**Table 1.** Characteristics of People Injured at Home

Characteristics	No. (%)
**Gender**	
Male	5915 (58.3)
Female	4231 (41.7)
**Place of residence**	
City	8864 (87.4)
Village	1282 (12.6)
**Age, y**	
0 - 4	1279 (12.6)
5 - 14	1826 (18)
15 - 24	1851 (18.3)
25 - 35	1154 (11.4)
35 - 44	877 (8.7)
45 - 54	677 (6.7)
55 - 64	582 (5.7)
65 ≤	1890 (18.6)
**Education**	
Diploma and higher	303 (3)
High school graduate	2075 (20.5)
Primary or secondary school	4330 (42.7)
Illiterate	1548 (15.3)
Children under 6 years old	1890 (18.6)
**Occupation**	
Housewife	2759 (27.2)
Farmer	133 (1.3)
Worker	1635 (16.1)
Staff	213 (2.1)
Unemployed	375 (3.7)
Other self-employed	1204 (11.9)
Students	1942 (19.1)
Children under 6 years old	1890 (18.6)

**Table 2.** Sex Distribution of Injury Mechanism

	Male, No. (%)	Female, No. (%)	*P* value
** **			< 0.001
**Fall from height**	3185 ( 53.8)	2424 (57.3)	
**Sharp objects injury**	1176 (19.9)	359 (8.5)	
**Fall on the level**	698 (11.8)	831 (19.7)	
**Building collapse**	259 (4.4)	206 (4.9)	
**Conflict**	244 (4.1)	152 (3.6)	
**Others a**	363 (6)	259 (6.1)	
**Total**	5915 ( 100)	4231 (100)	

^a^Electric shock, suicide, hand caught in a fan or foot caught in a bicycle wheel

There was a statistically significant difference between the sexes and the mechanism of injury (P < 0.001). Table 3 results show that the most common age for falling from a height and a fall on the same level in men was 5-14 year (22.8%, and 22.9%, respectively), but in females aged more than 65 years the rate was much higher (21.9%, 42.2% respectively).

**Table 3.** Age Distribution of Injury Mechanism by Sex

	Fall From Height, No. (%)		Fall on the Level, No. (%)		Building Collapse, No. (%)		Conflicts, No. (%)		Others, No. (%)	
**Age, y**	**Male**	**Female**	**Male **	**Female**	**Male**	**Female**	**Male**	**Female**	**Male**	**Female**
**0 - 4**	485 (15.3)	372 (15.3)	96 (13.8)	81 (9.7)	34 (13.1)	20 (9.7)	1 (0.4)	1 (0.2)	57 (16.2)	53 (20.5)
** 5 - 14**	725 (22.8)	331 (13.7)	160 (22.9)	110 (13.2)	67 (25.9)	35 (17)	17 (7)	0 (0)	114 (32.4)	40 (15.4)
** 15 - 24**	559 (17.6)	174 (7.2)	138 (19.8)	62 (7.5)	49 (18.9)	29 (14.1)	103 (42.2)	42 (27.6)	59 (16.8)	30 (11.6)
** 25 - 34**	309 (9.7)	187 (7.7)	57 (8.2)	58 (7)	29 (11.2)	21 (10.2)	63 (25.8)	41 (27)	38 (10.8)	21 (8.1)
** 35 - 45**	248 (7.8)	219 (9)	47 (6.7)	50 (6)	22 (8.5)	25 (12.1)	28 (11.5)	40 (26.3)	26 (7.4)	22 (8.5)
** 45 - 54**	230 (7.2)	193 (8)	30 (4.3)	56 (6.7)	17 (6.6)	11 (5.3)	18 (7.4)	16 (10.5)	13 (3.7)	25 (9.7)
** 55 - 64**	140 (4.4)	242 (10)	27 (3.9)	63 (7.6)	15 (5.8)	18 (8.7)	8(3.3)	6 (3.9)	12 (3.4)	12 (4.6)
65 ≤	481 (15.1)	706 (21.9)	143 (20.5)	351 (42.2)	26 (3.7)	47 (22.8)	6 (2.5)	6 (3.9)	33 (9.4)	56 (21.6)
***P * value**	< 0.001		< 0.001		< 0.002		< 0.001		< 0.001	

Older women (over 65 y) were injured 2.45 times more than older men in falls on the same level (42.5%, and 20.5% respectively). Also, the most common age for injury due to sharp objects and conflict was 15 - 24 years in both men and women. There was a statistically significant difference between the injury mechanism and sex in the all age groups (P < 0.001). Findings for the mechanism of injury by age group showed that falls (either from height or on the level) is the most common cause of home injury in all age groups. As shown in Table 4, limbs were the most common body region that was injured (73.7%) and the second region was the head and neck (23.6 %).

**Table 4.** Injured Body Region

Body Region	No. (%)
**Limb**	7477 (73.7)
**Head &Neck**	2404 (23.6)
**Spinal cord**	96 (0.9)
**Abdomen**	77 (0.7)
**Chest**	66 (0.6)
**Pelvic and Urinary system**	46 (0.4)
**External genitalia**	10 (0.1)
**Total**	10146 (100)

Table 5 indicates the outcome of home injuries during the study period. As shown, 0.4% of injuries resulted in death, 8.3% of injured patient had a complete recovery, 13% of patients required long-term follow-up, and 67.3% had a partial recovery. The most common cause of death from an injury was falling from a height (53.8%), followed by a fall on the level (33.3%), and building collapse (5.1%).

**Table 5.** Outcome of the Home-Related Injuries

Outcome	No. (%)
**Complete recovery**	844 (8.3)
**Partial recovery**	6827 (67.3)
**Long-term follow up**	1322 (13)
**Death**	39 (0.4)
**Unknown**	1114 (10.9)
**Total**	10146 (100)

Our results indicate that only 11.8% of patients were transported to the hospital by ambulance and 88.2% were transferred by non-special vehicle. 

## 5. Discussion

Study results show that 25.2% of all injuries in Kashan City were home injuries, which is compatible with findings from other reports (6, 10). This study showed that home injuries were more common in men than in women. This may be due to the greater involvement of Iranian men in daily home activities and it may also be due to men experiencing more severe injuries than women which need a hospital referral. This is similar to the findings of several studies in Iran, in that most of the patients with home injuries were men (13, 15, 16). But a study by Dianati et al. in Kashan City (2003) showed that the people most often injured were women (17). This difference may be due to sampling methods and the selection of people injured in a home setting and not a hospital setting. Our study showed that in the age group under 15 years, boys sustained more injuries than girls. This difference is possibly related to the fact that boys are more active than girls in the same age group and usually exposed to more accidents than girls. These finding are also similar with other studies in Iran, the UK and the USA (14, 15, 17-19). Older women had more injuries than men. This finding is compatible with results of two studies (14, 15), and may be related to the amount of exposure to the home environment between older men and women. A study on elderly people showed that 53% of home injuries victims were women and 47% were men (20). Results showed that falling from a height was the most common injury mechanism, followed by sharp object injuries and falls on the level. A study on elderly people in Izmir, Turkey, showed that a fall was the most common cause of home injury (61.8%), and the second most common cause (22.0%) was cut or piercing (21). Several studies also showed that the leading causes of home injuries are falls and being struck by objects (6, 13, 16, 18). However, in many studies burns were the most common injury mechanism (14, 15, 22). These differences may be related to the fact that in our hospitals, burn cases are admitted directly to burn units and they are not registered in trauma data bank. Fall injuries were more common in women than in men, but men experience more sharp object injuries and conflict than women. The results of Dianati’s study in Kashan were different from ours; falls were the most common in men (40.7%) and injuries by sharp objects (34.9%) were the most common in women (17). This difference may be due to the fact that their study was based on self-reported data, whereas our study was based on medical reporting systems. In our study, limbs were the most common body region that was injured, followed by the head and neck. In one study, 69% of patients had limb injuries and 12% had head or face injuries (15) which was similar to our study and somewhat consistent with the most common mechanism of injuries. A mortality rate of 0.4% for home injuries was seen in our study. Similarly, in a study on home injuries in Italy, the mortality rate was approximately 0.43% (23). Moreover, in some studies in Iran, this rate was near our rate (13, 15, 16), but in another study the mortality rate was greater than ours at about 1.3% (14). Delay in transferring the injured patient to hospital, especially in rural areas, a lack of suitable knowledge or practice of people in confronting injuries and differences in the delivery of medical services between communities may be reasons for these discrepancies. Our findings indicate that only 11.8% of trauma patients were transferred to the hospital by ambulance. The rate in the Fazel et al. study for all types of trauma was 40% and for home accidents it was 14% (11) which is similar to our figures. In comparison with other cities, our transportation rate by ambulance was higher (24, 25), but it is lower than in more developed countries. Thus, there must be a greater emphasis put on people’s education to use emergency medical services for the transportation of injured people to the hospital. The results of the present study indicate that a fall was the most common mechanism in home injuries. Therefore fall-related injury prevention programs should be designed to make homes safer and also focus on education about changes in life-style such as; diet, physical activity and smoking for reducing fall susceptibility. In addition, planning educational programs to increase people’s knowledge of how to deal with injuries is recommended.
